# Editorial: Neuro-immune interactions and neuroinflammation in neurocritical care

**DOI:** 10.3389/fimmu.2023.1147426

**Published:** 2023-01-30

**Authors:** Suyue Pan, Junlei Chang, Liping Liu, Xiaofeng Jia

**Affiliations:** ^1^ Department of Neurology, Nanfang Hospital, Southern Medical University, Guangzhou, China; ^2^ Institute of Biomedicine and Biotechnology, Shenzhen Institute of Advanced Technology, Chinese Academy of Sciences, Shenzhen, China; ^3^ Department of Neurology, Beijing Tiantan Hospital, Capital Medical University, Beijing, China; ^4^ China National Clinical Research Center for Neurological Diseases, Beijing, China; ^5^ Department of Neurosurgery, University of Maryland School of Medicine, Baltimore, MD, United States; ^6^ Department of Biomedical Engineering, The Johns Hopkins School of Medicine, Baltimore, MD, United States; ^7^ Department of Orthopedics, University of Maryland School of Medicine, Baltimore, MD, United States; ^8^ Department of Anatomy and Neurobiology, University of Maryland School of Medicine, Baltimore, MD, United States; ^9^ Department of Anesthesiology and Critical Care Medicine, The Johns Hopkins School of Medicine, Baltimore, MD, United States

**Keywords:** neuro-immune interactions, neuroinflammation, neurocritical care, gut microbiota, systemic immune responses

In recent years, great progress has been made in neuro-immune interactions, at the forefront of neurocritical care research. This Research Topic, Neuro-Immune Interactions and Neuroinflammation in Neurocritical Care, presents two original research articles and five reviews to highlight the mechanisms underlying systemic neuro-immune crosstalk and novel avenues for the prevention or treatment of severe neurological disorders.

## Role of diabetes in cerebral-cardiac syndrome after ischemic stroke

Cerebral-cardiac syndrome (CCS) can be launched by ischemic stroke and has been regarded as the second leading cause of death in post-stroke patients. Diabetes is strongly evidenced to correlate causatively with stroke, suggesting the close involvement of diabetes in CCS. Lin et al. provided an excellent overview of the characteristics of CCS and the potential role of diabetes in CCS. Diabetes induces the pre-CCS cardiac injury, triggers severe systemic inflammation after ischemic stroke, and therefore aggravates cardiac pathological damage of CCS. Of note, the NOD-like receptor pyrin domain containing 3 (NLRP3) inflammasome may act as a critical element of violent inflammation in diabetic CCS. However, further studies on the other pathogenesis of diabetic CCS are warranted.

## Mechanical ventilation management and risk factors for anti-N-methyl-D-aspartate receptor encephalitis

Patients with severe anti-N-methyl-D-aspartate receptor (NMDAR) encephalitis often require mechanical ventilation (MV). Lin et al. reported a prospective study of 305 patients with anti-NMDAR encephalitis. A total of 62 (20.3%) patients required MV, and 27 of them required prolonged MV (>15 days). Prolonged MV (*p* = 0.02), higher levels of C-reactive protein (*p* = 0.01), and neutrophil-to-lymphocyte ratio (*p* = 0.004) were found to be associated with poor neurological outcomes at 6 months. Notably, the majority of anti-NMDAR encephalitis patients with MV obtained a satisfactory long-term outcome after appropriate immunotherapy, despite the longer duration of MV.

## Influence of gut microbiota disorders on the prognosis of stroke

Recent studies have revealed that gut microbiota plays a pivotal role in the pathophysiological process of ischemic stroke. Wang et al. summarized the mechanisms underlying the interactions between the gut microbiota and the brain, and the impact of gut microbiota disorders on the pathogenesis of ischemic stroke. Gut microbiota disorders impact the post-stroke prognosis through microflora migration, intestinal bacterial metabolites, and immune regulation. The alterations in the gut microbiota have the potential as an indicator of ischemic stroke prognosis. Overall, gut microbiota regulation may represent a breakthrough in ischemic stroke therapy, and further prospective studies are needed for future clinical translation.

Secondary pulmonary infection is common after stroke and is associated with worse outcomes, while its mechanism and dynamic process remain obscure. Zhang et al. analyzed the changes in the immune system and intestinal barrier function in a mice model of hemorrhagic stroke. Dysfunctional immune system and impaired intestinal barrier function occurred rapidly after a hemorrhagic stroke, promoting the migration of enteric bacteria, which contributed to the secondary pulmonary infection. These findings highlight the importance of brain-gut crosstalk in poststroke pneumonia.

Taken together, these articles characterize the pivotal role of gut microbiota dysbiosis in deteriorated neurofunction and secondary peripheral organ dysfunction, paving the way to developing new proposals for the prevention or treatment of stroke.

## Connecting the brain to the periphery reciprocally through systemic immune responses after stroke

Ischemic stroke often causes multiple systemic complications, in which the systemic immune responses are involved in every stage. Wu et al. outlined the central-peripheral immune crosstalk after ischemic stroke in an excellent review. The neuroinflammation caused by ischemia-reperfusion injury rapidly triggers the systemic inflammatory cytokine storm and turns peripheral organs into the second battlefield of the post-stroke immune response. Meanwhile, the dysfunction of the HPA axis and autonomic nervous system after onset can evoke immune disorders and further impair peripheral organs. Eventually, the above signals feed back into the brain and form a vicious cycle, leading to an adverse prognosis.

Peripheral organ injury after stroke exacerbates brain damage and hinders neurofunction rehabilitation. Wang et al. presented a wonderful overview of its underlying pathophysiological mechanisms. Immune response links the post-stroke peripheral organ injury to the brain damage reciprocally. During the acute phase of stroke, strong immunosuppression in the peripheral organs can lead to peripheral organ infection and subsequent organ damage. The early detection and appropriate treatments of post-stroke peripheral organ injury may help improve the outcomes.

Wicks et al. systematically outlined the biology of microglia and monocyte-derived macrophages (MoDMs) and their roles in mediating the immune response to ischemic stroke in a review. Activated microglia and MoDMs are crucial mediators of the intracerebral inflammatory response *via* polarizing into pro- or anti-inflammatory phenotypes after ischemic stroke. Current studies mainly focus on switching the neuroimmune cells into anti-inflammatory phenotypes in acute and subacute phases of stroke and present promising avenues for treatment development. However, the ignorance of chronic inflammatory events following stroke hinders clinical translation.

Collectively, these reviews highlight the crucial role of systemic immune responses in the pathogenesis of stroke. After onset, the immune disorders ignited in the brain rapidly expand to peripheral organs and aggravate the brain injury in turn, in which the microglia and MoDMs in the proinflammatory phenotype act as a bridge.

## Summary

In summary, neuro-immune interactions and central-peripheral crosstalk have become research focuses currently. The articles in this Research Topic collection have presented novel insights on the impacts of immune responses and gut microbiota disorders on peripheral organ dysfunction and the post-stroke outcomes. After stroke onset, the launch of neuroinflammation and subsequent boosted systemic immune disorders mediate the interaction between the brain and peripheral organs, and continuously amplify adverse signals in the vicious cycle, in which the gut microbiota disorders further aggravate the multiple organ dysfunction and brain damage. The collection has also included the comprehensive analyses of cardiac pathological damage after stroke combined with metabolic disorders, and the risk factors of anti-N-methyl-D-aspartate receptor encephalitis patients requiring mechanical ventilation. Although further studies are still needed to transform these findings into clinical applications, this Research Topic collection will pave the way for developing targeted therapeutic strategies in neurocritical care clinical practice.

## Author contributions

SP, LL, and JC contributed to the drafting of this Editorial for the Research Topic that they edited. XJ made critical revisions and finalized the Editorial. All authors contributed to the article and approved the submitted version.

